# Can we send patients with large non-resolving pneumothorax home post chest drain removal?

**DOI:** 10.1186/1749-8090-10-S1-A190

**Published:** 2015-12-16

**Authors:** Sanjeet SA Singh, Karim Morcos, Alan JB Kirk

**Affiliations:** 1 Golden Jubilee National Hospital, Clydebank, Dumbartonshire, G81 4DY, UK

## Background/Introduction

In thoracic surgery, access to the pleural cavity involves a pleurotomy. A chest drain is inserted to allow re-expansion of the lungs post-pleurotomy. This also prevents a tension pneumothorax. Some patients have a residual pneumothorax post chest drain removal noted on chest radiography (CXR) despite having no air leaks. Rates of pneumothorax post chest drain removal vary with figures quoted at 9.3-13.6%. The majority of these are barely perceptible or small (<1 cm from pleural line to the apex of the hemithorax). Some are larger (>2 rib spaces in apex or base). For these larger pneumothoraces, is it safe to send patients home?

## Aims/Objectives

To assess the progress of patients discharged with large non-resolving pneumothoraces.

## Method

A retrospective observational study was done at our unit over a 6-month period. All patients had chest drains postoperatively and were discharged if there were no air leaks or worsening of their pneumothorax post drain removal. A repeat CXR was obtained during routine follow up 6 weeks later. Patients with pneumonectomies and permanent thoracostomies were excluded from the study. Air leaks were detected using digital drainage systems.

## Results

There were 158 patients in the study. The mean age was 59.7 years (SD = 16.6). All patients were asymptomatic at the time of discharge and none required further intervention in other hospitals with regards to their pneumothorax. There were 9 (5.7%) patients who were discharged with large residual pneumothorax (>2 rib space) visible on CXR. The mean age of this cohort was 69.7 years (SD = 8.8). During follow up, these residual spaces were either partially or fully fluid filled with no radiological or symptomatic worsening.

## Discussion/Conclusion

This study found that it was safe to discharge asymptomatic patients with a large pneumothorax provided they are haemodynamically stable and had no air leak.

